# Compact Frequency-Agile and Mode-Reconfigurable Antenna for C-Band, Sub-6-GHz-5G, and ISM Applications

**DOI:** 10.3390/mi16060724

**Published:** 2025-06-19

**Authors:** Esraa Mousa Ali, Wahaj Abbas Awan, Anees Abbas, Syed Mujahid Abbas, Heba G. Mohamed

**Affiliations:** 1 Faculty of Engineering, Communications and Computer Engineering Department, Al-Ahliyya Amman University, Amman 19111, Jordan; 2 Department of Information and Communication Engineering, Chungbuk National University, Cheongju 28644, Republic of Korea; 3 Department of Mechanical Engineering, Yokohama National University, Yokohama 240-0067, Japan; 4 Department of Electrical Engineering, College of Engineering, Princess Nourah bint Abdulrahman University, P.O. Box 84428, Riyadh 11671, Saudi Arabia; 5 Electrical Department, College of Engineering, Alexandria Higher Institute of Engineering and Technology, Alexandria 21421, Egypt

**Keywords:** compact antenna, frequency reconfigurability, 5G-sub-6GHz, ISM band, smart electronics

## Abstract

This article presents the design and evaluation of a compact-sized antenna targeting heterogenous applications working in the C-band, 5G-sub-6GHz, and the ISM band. The antenna offers frequency reconfigurability along with multi-operational modes ranging from wideband to dual-band and tri-band. A compact-sized antenna is designed initially to cover a broad bandwidth that ranges from 4 GHz to 7 GHz. Afterwards, various multiband antennas are formed by loading various stubs. Finally, the wideband antenna along with multi-stub loaded antennas are combined to form a single antenna. Furthermore, PIN diodes are loaded between the main radiator and stubs to activate the stubs on demand, which consequently generates various operational modes. The last stage of the design is optimization, which helps in achieving the desired bandwidths. The optimized antenna works in the wideband mode covering the C-band, Wi-Fi 6E, and the ISM band. Meanwhile, the multiband modes offer the additional coverage of the LTE, LTE 4G, ISM lower band, and GSM band. The various performance parameters are studied and compared with measured results to show the performance stability of the proposed reconfigurable antenna. In addition, an in-depth literature review along with comparison with proposed antenna is performed to show its potential for targeted applications. The utilization of FR4 as a substrate of the antenna along with its compact size of 15 mm × 20 mm while having multiband and multi-mode frequency reconfigurability makes it a strong candidate for present as well as for future smart devices and electronics.

## 1. Introduction

The evolution of communication systems over the past few decades has fundamentally transformed how we connect, share information, and access services. This progress has been further amplified by the advent of the Internet of Things (IoT), which has brought about a paradigm shift in the number of devices requiring connectivity [1,2,3]. The IoT is a cornerstone of modern wireless communication, fostering an interconnected world with intelligent, efficient, and scalable networks. It enables a connected ecosystem of devices, sensors, and systems [4]. The IoT leverages modern wireless technologies to facilitate seamless data exchange, automation, and intelligent decision-making across various domains, including smart homes, healthcare, agriculture, and industrial automation [5,6]. As wireless communication evolves toward 6G and beyond, the IoT will remain at the forefront of technological innovation, driving the future of connectivity [7].

The rapid advancements in communication systems, coupled with the rise of the IoT, are redefining the expectations for handheld devices. The increased reliance on wireless services for seamless communication and data transfer has made it essential for modern devices to integrate multiple functionalities efficiently [8,9]. Antennas, being critical components of wireless devices, need to adapt to these varying demands. This is driven by the increasing complexity of wireless devices, which must operate across multiple bands, support diverse applications, and be adaptable to changing environments [10,11]. Reconfigurable surfaces are emerging as a potential solution for the aforementioned problems; however, their size and biasing complexity along with the power required are the challenges that need to be tackled [12,13,14]. Incorporating reconfigurability into antenna designs has emerged as a promising solution to these challenges by incorporating the ability to dynamically adjust their operating characteristics to meet specific system needs. Reconfigurable antennas can adjust their frequency, radiation pattern, or polarization to maintain optimal performance across different conditions [15,16,17,18].

Frequency-reconfigurable antennas are a class of antennas that are capable of dynamically altering their operating frequency to support different communication bands. Thus, they mitigate the need for additional filters to stop the unnecessary bands [19,20]. Moreover, these antennas are integral to modern wireless systems, which must operate across multiple frequency bands to accommodate various communication standards and applications such as 5G, LTE, the IoT, and satellite communication [21,22,23,24]. The integration of frequency-reconfigurable functionality into a single compact structure eliminates the need for multiple fixed antennas, making them ideal for space-constrained devices like smartphones, wearables, and IoT sensors, thus reducing the manufacturing and design complexity. Frequency reconfigurability in antennas is achieved through various mechanisms that dynamically adjust the antenna’s structure, electrical properties, or material characteristics. These mechanisms mainly involve switch-based reconfiguration (using PIN diodes and MEMS switches) [25,26], tuning component-based reconfiguration (using Varactor diodes) [27,28], material-based reconfiguration [29,30], as well as mechanical alteration of the antenna geometry to achieve frequency reconfigurability [31].

Among the above-mentioned techniques for frequency reconfigurability, PIN diodes are widely used in antenna designs due to their characteristics such as their fast switching, reliability, and ease of integration. By acting as electronic switches, they enable dynamic changes in the antenna’s operating frequency to meet the requirements of modern wireless systems. Moreover, PIN diodes are inexpensive compared to other switching technologies like MEMS. PIN diodes consume a relatively low amount of power when switching states, especially in comparison to continuously active tuning components like Varactor, which makes them suitable for battery-operated devices, such as IoT sensors and mobile communication systems. Lately, various studies have been reported in the literature where frequency reconfigurability has been achieved using PIN diodes [21,22,26,32,33,34] for multiple wireless communication applications. In [21], a Substrate-Integrated Waveguide (SIW)-based antenna supporting S-band and C-band applications is proposed, for which frequency reconfigurability is achieved by using several PIN diodes. Although reconfigurability for two modes of operation is achieved for this design, use of several PIN diodes as well as the SIW increases the complexity of this design.

The authors in [22] reported a frequency-reconfigurable monopole antenna for 5G Sub-6-GHz, and WLAN applications. Four different modes were achieved by employing three PIN diodes. Another frequency-reconfigurable monopole antenna operating in the microwave frequency band is presented in [26]. This design involves four PIN diodes to attain five modes of operation. Although this design succeeds in achieving five modes, the geometrical dimensions of this structure are comparatively large. An inverted F-shaped antenna with frequency-reconfigurable characteristics is proposed in [32]. Frequency reconfigurability is achieved by using two PIN diodes. This antenna resonates between 0.841 GHz and 2.12 GHz while covering six different frequency bands. In addition, the authors in [33] proposed a multilayered antenna structure for S-band and C-band wireless communication applications, where frequency reconfiguration was attained by incorporating four PIN diodes. Likewise, in [34], a frequency-reconfigurable antenna for WiMAX and Wi-Fi applications is presented. For this design, frequency reconfigurability is achieved through a PIN diode and allows switching between two different frequency bands. This antenna exhibits comparatively larger dimensions.

**Figure 1:**
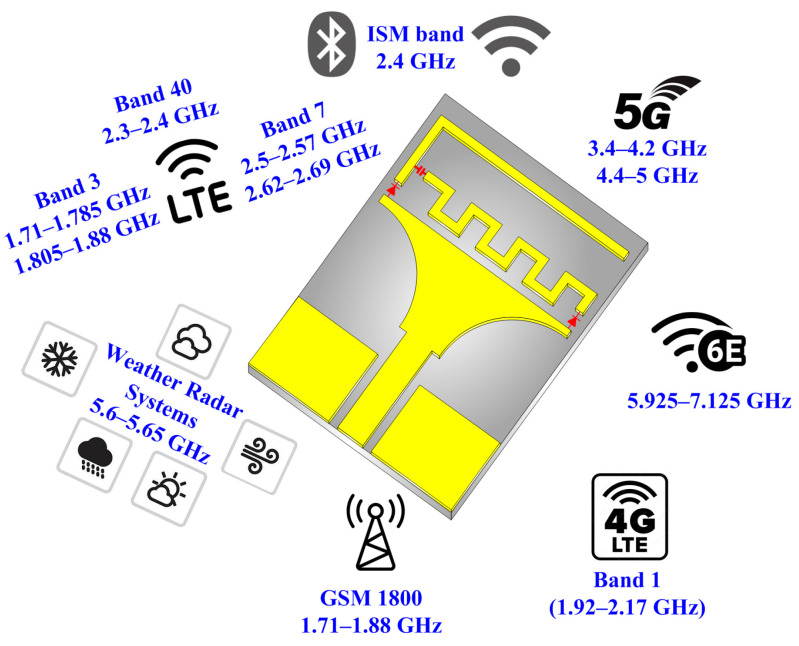
Targeted applications of proposed reconfigurable antenna.

Frequency-reconfigurable antennas are a cornerstone of modern wireless communication, enabling efficient, flexible, and adaptive connectivity. Therefore, this article proposes a compact-sized antenna with multiband operation for IoT applications. The frequency-reconfiguration capability is attained by using two diodes, where four distinct operational modes are achieved. Serpentine-shaped stubs are introduced to achieve compactness. The proposed antenna supports C-band, 5G-sub-6-GHz, Wi-Max, ISM-band, GSM, and LTE applications, as depicted in Figure 1.

## 2. Antenna Configuration and Design Strategy

### 2.1. Antenna Design

**Figure 2:**
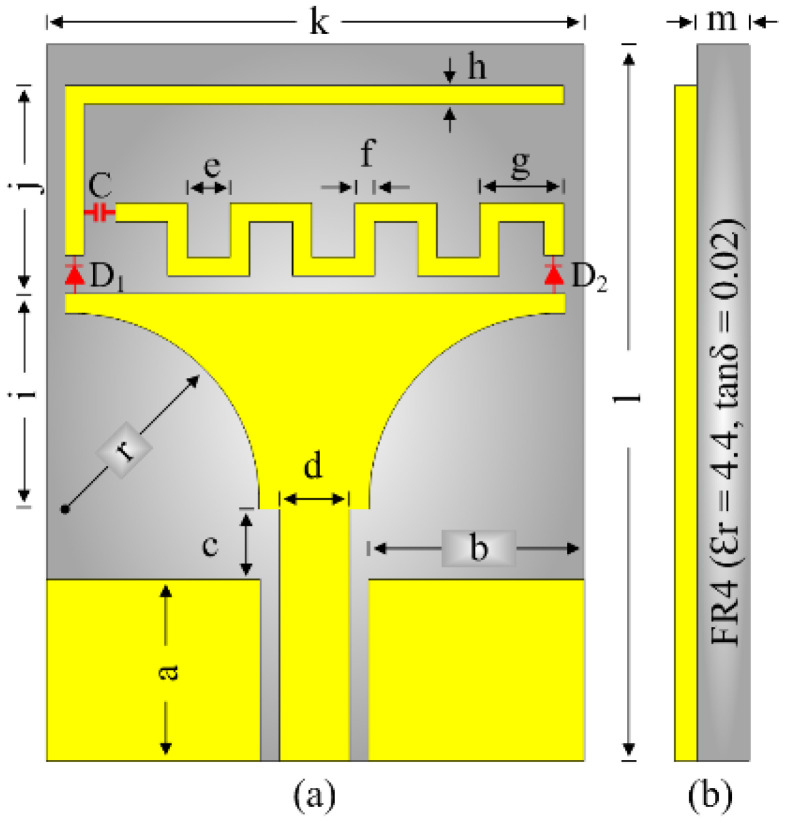
Proposed multi-mode frequency-reconfigurable antenna: (**a**) top view, (**b**) side view.

Figure 2 illustrates the design configuration of the proposed reconfigurable antenna. The antenna comprises a co-planar waveguide (CPW)-fed truncated-shaped radiator, which is further connected to an inverted L-shaped stub by means of an HPND-4005 diode D_1_. Another meandered line stub is also connected to the top-right edge of the radiator by using a PIN diode D_2_. A 100-pF surface-mounted (SMD) capacitor is placed at the node connecting both stubs to electrically separate them, so that each stub can work independently depending upon the switching state of the diode. The antenna offers a compact size of 15 × 20 mm^2^, whilst being easily accessible, and the cheap printed circuit-board material FR4, with a relative permittivity of 4.4 and thickness of 1.6 mm, is utilized as the substrate. The optimized dimensions of the proposed antenna and their respective values are as follow: a = 5; b = 6; c = 2; d = 2; e = 1.5; f = 0.5; g = 2.5; h = 0.5; i = 6; j = 6.4; k = 15; l = 20; m = 1.6, and r = 5.5 (unit is mm).

### 2.2. Systematic Design Process

The proposed frequency-agile antenna is extracted from the conventional CPW printed antenna by following two major steps. Step 1 includes the designing of a compact antenna to operate at multiple frequency bands, depending upon the presence of the stub along with the main radiator. Step 2 focuses on achieving frequency reconfiguration without affecting the overall size of the antenna along with the multi-mode operation.

#### 2.2.1. Multiband Antenna

**Figure 3:**
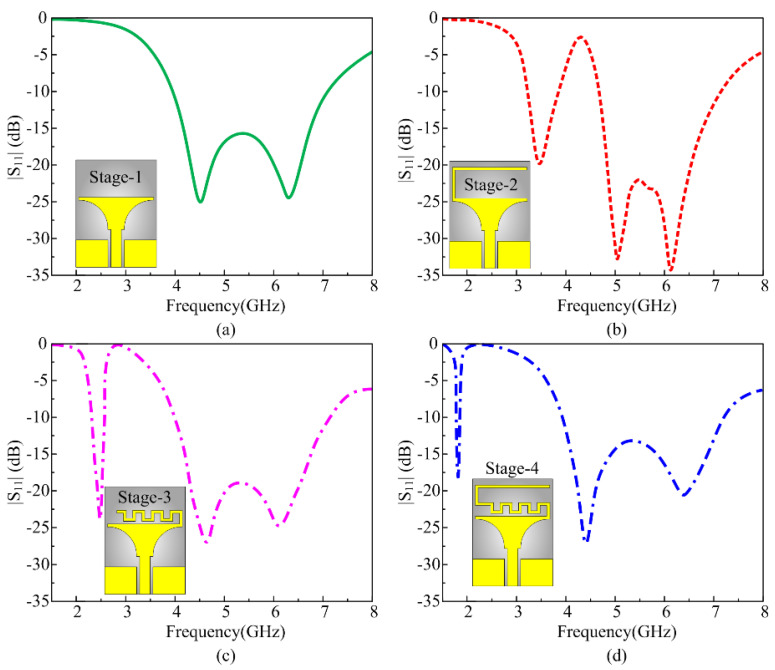
Proposed multi-mode frequency-reconfigurable antenna: (**a**) top view, (**b**) side view; (**a**) stage 1, (**b**) stage 2, (**c**) stage 3, (**d**) stage 4.

Antenna design passes through various consecutive iterations to achieve the multiband operation. For the said purpose, a truncated printed structure is selected as the base of the design, as depicted in Figure 3a. Truncated structures are well known for their broadband behavior, and the smooth transition of the shape from the rectangular feed toward the radiator allows more current to flow, which consequently improves the overall bandwidth of the antenna. The resultant antenna in stage 1 possesses the wideband ranges from 3.85 GHz to 7.37 GHz, as depicted in Figure 3a. Hereafter, the stubs are loaded to achieve multiband behavior, and efforts are made to achieve lower bands across 1.8 GHz. For this objective, initially an inverted L-shaped stub is loaded at the top-left side of the radiator, as shown in the inset of Figure 3b. The stage-2 antenna offers the dual-band response while covering the lower band ranges from 3.27 GHz to 4.02 GHz, while the upper band ranges from 4.72 to 7.29 GHz, as depicted in Figure 3b. The lower band is the result of loading the stub, whereas the effective length of the stub to achieve the lower band is calculated by using the following expression provided in [35,36]:

(1) $$f_{L}=\frac{c}{xL_{e}\sqrt{\varepsilon_{eff}}}$$

Here, the f_L_ shows the resonating frequency due to addition of the stub, which is the ratio of the speed of light in a vacuum (c) to the product of the effective length of the stub (L_e_), the square root of the effective permittivity (ε_eff_) of the substrate, and variable x, which correspond to the fraction of the wavelength at the desired frequency f_L_. Rearranging the Equation (1) results in the following expression, which is utilized to estimate the length of the stub for the desired frequency of 3.5 GHz:

(2) $$L_{e}=\frac{c}{xf_{L}\sqrt{\varepsilon_{eff}}}$$

By inputting the value, the effective length is mm with x corresponding to 4.

Following the same aforementioned methodology, a meandered line stub is loaded to the top of the radiator in stage 3, as depicted in the inset of Figure 3c. The stub consequently achieves resonance at 2.45 GHz with an impedance bandwidth of 2.1 GHz–2.6 GHz while the upper band remains unvaried, offering wideband behavior from 4.72 GHz to 7.29 GHz, as shown in Figure 3c. In the last stage, a combination of the stub designed earlier is utilized, where the inverted L-shaped structure is connected to the one end of the meandered line stub. This stage 4 effectively results in achieving the targeted band spectrum of 1.8 GHz ranging from 1.76 to 1.85 GHz, that covers the globally allocated band spectrum for GSM 1800 MHz applications. It is worth mentioning that the proposed antenna achieved the lower band of 1.8 GHz with physical size of 15 × 20 mm^2^ that corresponds to 0.09 λ_fl_ × 0.12 λ_fl_, where λ_fl_ is the wavelength at the lowest resonance.

#### 2.2.2. Multi-Mode Reconfigurable Antenna

**Figure 4:**
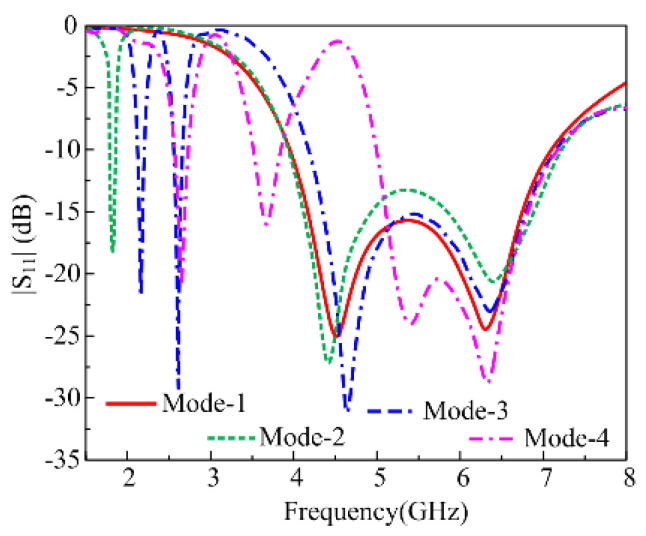
Return loss graph of various reconfigurable modes achieved by proposed antenna.

It is clear from the multiband antenna design stages that the two stubs and their unique combination can generate an additional lower stub while maintaining the wideband behavior of the radiator. Therefore, two PIN diodes are utilized to electrically connect and disconnect the stubs depending upon the user requirement. However, the diode can be triggered on or off simultaneously due to a physical connection, which is result of the stage-4 design. This arising problem is resolved by adding an additional capacitor at the junction of the stubs, as shown in Figure 2. The additional capacitor as well as the RLC load introduced by the addition of the diodes results in increased mismatching along with the shift in the lower frequency. Thus, optimization is performed by using a parametric analysis and the fine-tuned reconfigurable antenna offers four operational modes, as shown in Figure 4.

Mode 1: In this mode, both diodes are in the off state, which results in the generation of wideband ranges of 3.9–7.15 GHz.

Mode 2: In this mode, the diode D1 is switched off while keeping the D2 switch on, and a dual-band is achieved having a corresponding bandwidth of 1.78–1.84 GHz and 3.91–7.23 GHz.

Mode 3: This mode is in contrast to mode 2 where diode D2 is off while the diode D1 is switched on. Tri-band behavior is observed with the respective frequency ranges of 2.1–2.19 GHz, 2.54–2.65 GHz, and 4.2–7.2 GHz.

Mode 4: In this case, both diodes are switched on, which consequently results in the generation of another tri-band mode. Two additional lower resonances at 2.65 GHz and 3.65 GHz are achieved with respective bandwidths of 2.55–2.72 GHz and 3.5–3.85 GHz. Alongside that, wideband ranges from 4.99 to 7.15 GHz are achieved. Thus, the proposed antenna offers the advantage of having a compact size along with frequency reconfigurability by achieving three operational modes: the wideband mode, dual-band mode, and tri-band mode.

## 3. Antenna Testing and Result Analysis

### 3.1. Fabricated Prototype and Measurement Setup

**Figure 5:**
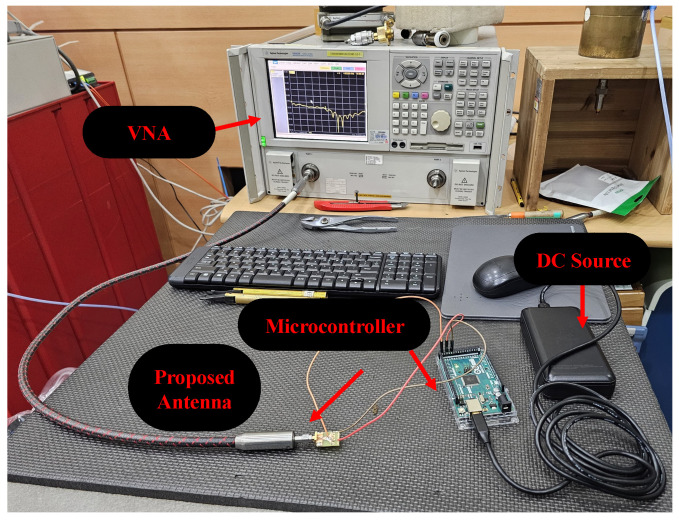
Measurement setup for scattering parameters.

Figure 5 illustrates the fabricated prototype of the proposed reconfigurable antenna under test conditions for return loss results. The measurement setup consists of four major parts:

(1) a high-performance portable network analyzer (PNA) by KEYSIGHT with the model number E8362B, with an operational frequency range of 10 MHz to 20 GHz.
(2) a proposed reconfigurable antenna along with soldered diodes, wires, and SMA connector.
(3) a microcontroller utilized to trigger the state of the diodes; an interval of 2 min is given to measure all of the switching states.
(4) A DC source that gives power to the microcontroller, which further provide controlled power to the diodes.

**Figure 6:**
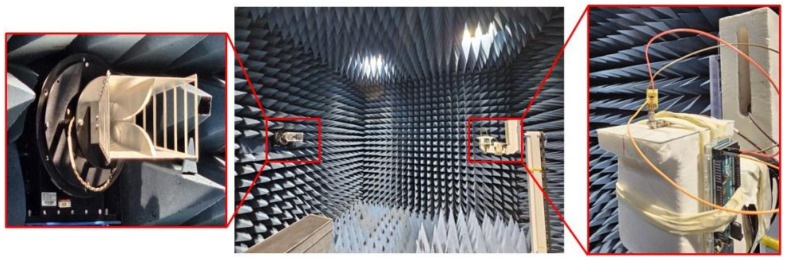
Far-field measurement setup for the proposed antenna system.

Figure 6 depicts the antenna under test conditions for far-field parameters. The antenna is tested inside the electromagnetic anechoic chamber, where the whole biasing network is also connected with the antenna to obtain the real-scenario results, as depicted in Figure 6. All measurements are carried out at Korea Radio Promotion Association (RAPA) internet-of-things (IoT) technical support center. In the forthcoming section, the performance parameters of each mode are presented to understand the behavior of the antenna for various switching states.

### 3.2. Mode 1 (Both D_1_ and D_2_ Are Off)

**Figure 7:**
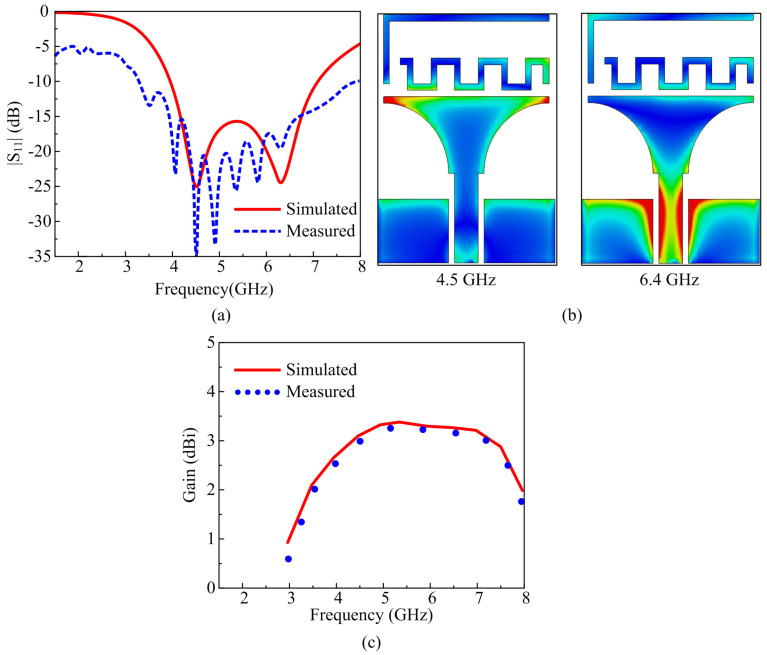
Mode 1 performance parameter comparison of (**a**) return loss, (**b**) surface current, and (**c**) peak gain.

In mode 1, when both diodes are in the off state, that corresponds to the working of the lower part of the radiator while the stubs do not play any role in the radiation. The proposed antenna offers a wideband mode, having an S11 < −10 dB simulated impedance bandwidth of 3.9–7.15 GHz, while the measured results offer 3.2–8 GHz, as depicted in Figure 7a. The measured bandwidth is wider as compared to the simulated results, which is due to the presence of the biasing cables that consequently induce additional coupling and affect the performance of antenna. To understand the working behavior of the antenna for mode 1, the surface current graphs at 4.5 GHz and 6.4 GHz are presented, as shown in Figure 7b. At both frequencies, the maximum current flows across the lower part of the radiator and CPW feed. Moreover, due to the close presence of the upper part of the radiator, i.e., stubs, a small amount of current is also induced in them; however, the performance remains unaffected as compared to the antenna without any diodes. Lastly, the gain over the operational wideband is also presented in Figure 7c, and the antenna offered a gain of more than 2.5 dBi, while the measured results also offered the strong comparison as compared with the simulated results. This wideband mode targeted the frequency band spectrum globally that was allocated for C-band applications along with coverage of the sub-band, but this was not limited to Wi-Fi, the ISM upper band, Wi-Fi 6E, and the weather radar system.

### 3.3. Mode 2 (D_1_ Is Off While D_2_ Is On)

**Figure 8:**
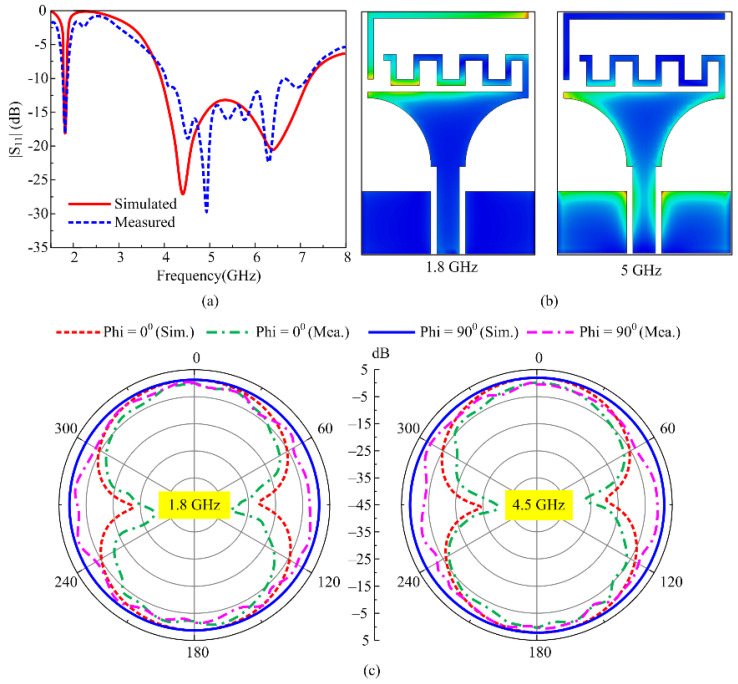
Mode 2 performance parameter comparison of (**a**) return loss, (**b**) surface current, and (**c**) peak gain.

In mode 2, corresponding to D_1_ being in the off state while D_2_ is in the on state, the antenna system offers a dual-band operational mode. Besides the wideband generated in mode 1, the antenna offers a low band across 1.8 GHz. The results offer corresponding simulated and measured values of 1.78–1.84 GHz and 1.77–1.87 GHz across the lower resonance. Meanwhile, at a higher resonance, the bandwidth of 3.91–7.23 GHz is observed for the simulated case, while the measured value ranges from 4.17 GHz to 7.13 GHz, as depicted in Figure 8a. The operational behavior can be understood by using the surface current graphs, where at a lower resonance of 1.8 GHz the maximum current is observed across the inner stub and upper part of the main radiator; moreover, a handsome amount of current is also induced in the outer stub as well, as depicted in Figure 8b As is depicted for mode 1, the higher bands are generated due to the main radiator and this is also validated by analyzing the surface current graph at 5 GHz.

Lastly, the radiation pattern of the antenna working in mode 2 at the selected frequencies of 1.8 GHz and 4.5 GHz is presented in Figure 8c. In both simulated and measured cases, the antenna offers well-matched results having an omni-directional radiation pattern for the Phi = 90° plane, while a monopole-like bi-directional radiation pattern is observed for the Phi = 0° plane, as depicted in Figure 8c. The antenna offers a peak gain of 1.2 dBi at 1.8 GHz, while a peak gain of 2.4 dBi is observed for 4.5 GHz. Mode 2 targets the band spectrum allocated for GSM, Bluetooth, LTE Band-3, 5G sub-6-GHz, Wi-Fi-6E and C-band applications.

### 3.4. Mode 3 (D_1_ Is On While D_2_ Is Off)

**Figure 9:**
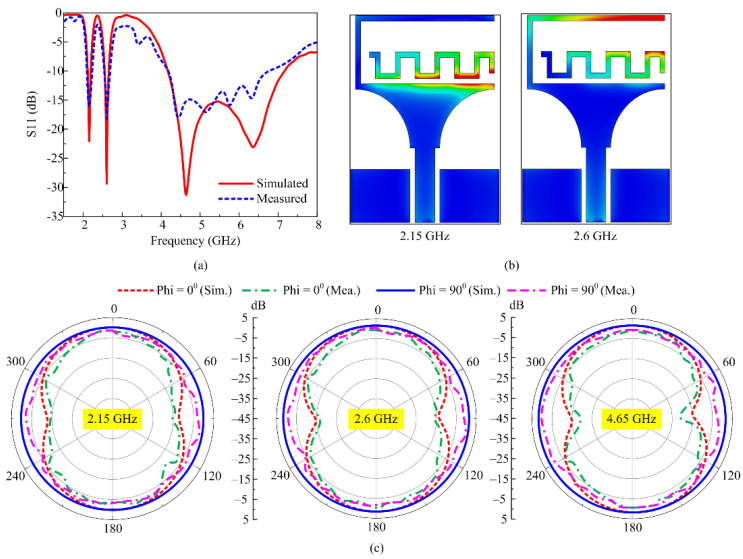
Mode 3 performance parameter comparison of (**a**) return loss, (**b**) surface current, and (**c**) peak gain.

In mode 3, when D_1_ is switched on while keeping D_2_ switched off, the antenna offers the tri-band behavior with wideband behavior similar to mode 1, and two distinguished resonances across 2.15 GHz and 2.6 GHz, as depicted in Figure 9a. Simulated results offer the impedance bandwidths of 2.1–2.19 GHz, 2.54–2.65 GHz, and 4.2–7.2 GHz, which correspond to 4.18%, 4.23%, and 64.5%, respectively. On the other hand, the measured result offers an S11 < −10 dB bandwidth of 2.09–2.21 GHz, 2.55–2.69 GHz, and 4.25–6.87 GHz, as depicted in Figure 9a. The surface current distribution is also studied, where at 2.6 GHz, the maximum current is concentrated in the outer stub, while a small amount of current is also induced in the inner stub. Contrary to that, at 2.15 GHz, the maximum current is observed in the inner stub, which is due to coupling among the stub and the radiator, as depicted in Figure 9b. Since the inner stub offers the bigger electric length, the coupling generates a lower frequency of 2.15 GHz, unlike the generation of 2.6 GHz due to the outer stub, which offers the smaller electrical length.

A comparison among the radiation pattern of the antenna at the selected frequencies of 2.15 GHz, 2.6 GHz, and 4.65 GHz is also illustrated in Figure 9c. For all frequencies, an omni-directional radiation pattern is observed for Phi = 90°, while a monopole-like radiation pattern is observed for Phi = 0°. For all frequencies, a strong comparison among simulated and measured results is offered, which validates the working of mode 3, as depicted in Figure 9c. Moreover, for the lower band, the antenna offers a gain of more than 1.1 dBi, while for the upper band, the gain is observed to be greater than 2.5 dBi. Furthermore, in mode 3, the proposed antenna covers the globally allocated band spectrum for 3G, LTE-band-7, LTE-4G-band-1/band-4/band-66, 5G-sub-6-GHz, Wi-Fi-6E, and C-band applications.

### 3.5. Mode 4 (Both D_1_ and D_2_ Are On)

**Figure 10:**
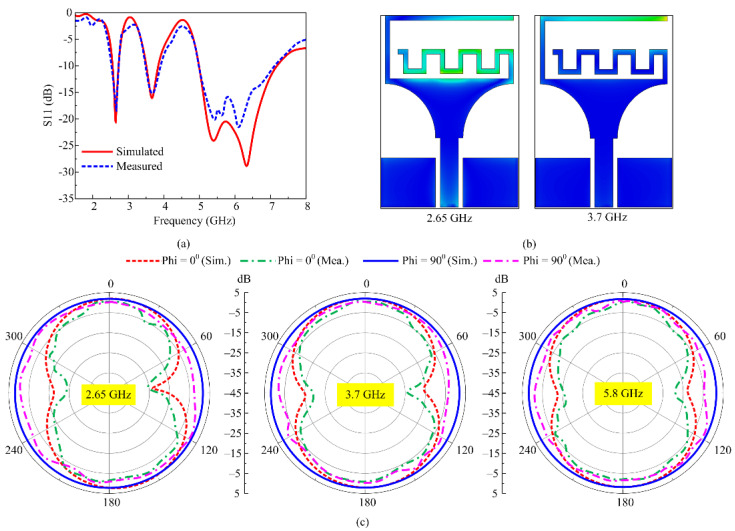
Mode 4 performance parameter comparison of (**a**) return loss, (**b**) surface current, and (**c**) peak gain.

Mode 4 corresponds to both diodes being in the on state. In this mode, the antenna offers a tri-band mode, where the simulated results show the S11 as being less than −10 dB with impedance bandwidth ranges of 2.55–2.72 GHz, 3.5–3.85 GHz, and 4.99–7.15 GHz, as shown in Figure 10a. The measured results also offer the tri-band mode, where the two narrow bands are observed across the resonance of 2.65 GHz and 3.7 GHz with respective bandwidths of 2.51–2.7 GHz and 3.47–3.9 GHz. On the other hand, the wideband ranges from 5.05 GHz to 7 GHz, as depicted in Figure 10a. The current distribution graphs are also presented for both lower band resonances of 2.65 GHz and 3.7 GHz, as shown in Figure 10b. It is clear from the current distribution analysis that the middle band of 3.7 GHz is generated due to the inverted L-shaped stub, as most of the current is observed across it. Meanwhile, the lower resonance of 2.65 GHz is due to the inner meandered line stub, which corresponds to a larger electrical size, as depicted in Figure 10b.

Surface current distributions of the antenna working in mode 4 are depicted in Figure 10c. For all of the selected frequencies of 2.65 GHz, 3.7 GHz, and 5.8 GHz, the antenna offers a monopole-like dual-beam radiation pattern in the principal E-plane (phi = 0°). On the other hand, for the principal H-plane (phi = 90°), the antenna offers a nearly omni-directional pattern. Moreover, in all cases, a good comparison among simulated and measured results is observed, with a slight deviation being due to the presence of biasing wires and other biasing components. Furthermore, the antenna offers the measured peak gain of 1.83 dBi at 2.65 GHz, while for 3.7 GHz, the peak gain of 2.11 dBi is observed along with gain of more than 2.5 dBi for the wideband range. Mode 4 covers the globally allocated band spectrum for LTE-Band-7, 5g-sub-6GHz, C-band, ISM-upper-band, Wi-Fi-6E, and weather radar system applications.

For all diode switching modes, the strong comparison among simulated and measured results validates the performance stability as well as the significance of the work. Moreover, the switching option among the wideband, dual-band, and tri-band modes, while covering the globally allocated band spectrum for various applications, raises the potential of the proposed antenna to be used in present and future compact smart electronics. Moreover, the simplified structure will minimize the fabrication tolerance while the measurement using the whole biasing structure strengthens its potential for practical application where multi-mode frequency reconfigurability is required.

### 3.6. Comparison with State-of-the-Art Technology

The proposed multiband multi-mode reconfigurable antenna is compared with the results of a recently published study targeting similar applications, as depicted in Table 1. Apart from that reported in [28], the proposed antenna offers a more-compact size. Although the antenna presented in [28] offers a smaller size it has the drawback of having a single-band operation while utilizing four PIN diodes. On the other hand, the antennas reported in [15,22,23] offer a multiband mode but they suffer from a narrow bandwidth and large electrical size. The antenna reported in [14] not only offers single-band reconfigurability but it also utilizes 12 PIN diodes with a large-sized radiator. Thus, it can be concluded that the proposed antenna offers the best solution, having the advantages of not being limited to a compact size and having multi-mode reconfigurability, while using two PIN diodes with a simple configuration, that increase its potential to be used in present and future compact electronic devices.

## 4. Conclusions

This research focuses on the design of a multiband antenna capable of achieving a multi-mode response to meet the requirements of modern-day applications. A compact-sized antenna with a physical size of 15 mm × 20 mm, corresponding to an electrical size of 0.09 λ_f_ × 0.12 λ_f_, was designed using the common PCB material FR4. Initially, a truncated structure was utilized to achieve a wideband response of 3.85–7.37 GHz. Afterward, the various-shaped stubs were loaded to achieve additional low bands of 1.8 GHz, 2.2 GHz, 2.5 GHz, and 3.5 GHz. The frequency reconfigurability was achieved using two PIN diodes, whereas the independent control of the diodes results in the multi-mode operation of wideband, dual-band, and tri-band modes. The dual-band mode covers the lower frequency band of 3.85–7.37 GHz and 3.85–7.37 GHz along with the wideband achieved using the initial design, whereas the tri-band covers the band spectrums of 3.85–7.37 GHz, 3.85–7.37 GHz, and 3.85–7.37 GHz. These frequency band spectrums cover the globally allocated band spectrum for GSM 1800 MHz, LTE (Band 3, Band 7, and Band 40), LTE-4G (Band 1), 5G-sub-6-GHz (lower and upper bands), WiFi (802.11a, 802.11n, 802.11ac, and 802.11ax), and WiFi-6E-band applications. Moreover, the comparison with the results of recently reported studies further highlights the potential of the proposed antenna, which is characterized by having multiple advantages, including its compact size, frequency reconfigurability, and multi-mode operation as well as a strong comparison among the numerical and tested results, indicating its suitability for smart electronics and modern-day devices that operate as multiband antennas.

## Figures and Tables

**Table 1:** Comparison of proposed reconfigurable antenna with antennas proposed in recent reported studies.

Ref.	Dimensions (mm × mm)	Reconfigurable Modes	Bandwidth (GHz)	No. of Switches
[14]	53.5 × 53.5	Single Band	2.4–2.42|5.68–6	12
[15]	30 × 20	Wideband	3.51–8.51	3
		Single Band	3.10–4.11	
		Dual Band	2.41–2.81|5.47–7.18	
		Tri-Band	2.03–2.27|4.61–5.35|5.87–7.22	
[16]	16 × 22	Single Band	5.15–5.27	2
		Tri-Band	2.14–2.17|4.02–4.47|5.15–5.53	
			2.08–2.11|3.13–3.2|4.49–5.2	
[21]	24 × 51	Single Band	0.85–1.3 *	1 **
[22]	34 × 34	Penta-Band	1.8|3.3|4.8|5.5|5.8 ^+^	4
		Hexa-Band	1.3|2.1|2.8|3.9|4.8|5.8 ^+^	
		Tri-Band	2.5|3.9|4.8 ^+^	
[23]	22 × 32	Octa-Band	2.34–2.82|3.84–4.53|5.61–5.985|10.46–12.84|13.84–14.50|15.69–17.37|18.21–19.86	1
		Penta-Band	3.84–4.53|5.61–5.985|7.89–8.79|13.84–14.50|15.69–17.37	
[25]	120 × 60	Single Band	0.96|2.1|2.12 ^+^	2
[28]	10 × 25	Single Band	0.698–0.96|1.6–2.6|3.4–3.8|5–6 *	4
Proposed	15 × 20	Wideband	3.9–7.15	2
		Dual Band	1.78–1.84|3.91–7.23	
		Tri-Band	2.1– 2.19|2.54–2.65|4.2–7.2	
			2.55–2.72|3.5–3.85|4.99–7.15	

* Total Frequency Range, ** Varactor diode, ^+^ bandwidth is not given.

## Data Availability

All data is included in the manuscript.
